# Mapping the road to better sleep: forecasting sleep quality using actigraphy-based machine learning hours before bedtime

**DOI:** 10.1093/sleep/zsaf148

**Published:** 2025-05-31

**Authors:** Ankit Parekh

**Affiliations:** Division of Pulmonary, Critical Care, and Sleep Medicine, Department of Medicine, Icahn School of Medicine at Mount Sinai, New York, New York, USA; Department of AI and Human Health, Icahn School of Medicine at Mount Sinai, New York, New York, USA

Sleep is an important, modifiable lifestyle behavior, on par with physical activity [1]. The current era marks a golden age for mobile health (mHealth) technology, particularly using physical activity trackers. These trackers largely aim to summarize one’s pattern of activity and leave it to the user to interpret the data. While there exists a segment of devices that provide insights in the form of “nudges” or “coaching” using the summary data acquired, they fall short of providing personalized recommendations. Further, they do not track the follow-through on those recommendations which can truly modify behavior and overall health [2–5]. A physical activity tracking system that can provide actionable insights prior to the adverse outcome, for example, poor sleep, would truly allow us to test the impact of personalized sleep recommendations on overall well-being. In sum, the holy grail of mHealth technology is achieving the current state of the global positioning system-based navigation system: we know the origin (daily activity patterns), we know the destination (optimal health), and expect that the system can dynamically adapt to detours (poor sleep) rather than serve as a static map simply showing us where we have been.

In this issue of SLEEP, Sakai et al. [6] use multi-night actigraphy data to predict sleep quality, defined as actigraphy-based sleep efficiency. The premise of the study is to utilize five days of accelerometer activity to predict sleep efficiency on the sixth night (final night) as low versus high using a cutoff of 0.85, 4, and 8 hours prior to sleep onset on the final night. The authors analyzed a total of 80,811 middle-aged and older adults from the UK Biobank study for their two machine-learning models. Of the entire cohort of analyzed subjects, approximately 23% had low sleep efficiency on their sixth night of sleep. The authors show that the developed models achieve high accuracy (median area under the receiver operating characteristic curve [AUROC]  > 0.9, and median area under precision-recall curve [AUPRC] > 0.79) in predicting low sleep efficiency 4 and 8 hours prior to bedtime.

The authors must be commended on developing two types of prediction models (and two variations based on either a 4-hour or 8-hour prior to bedtime prediction) and testing them through repeated k-fold cross-validation. The first model was a decision-tree-based model in which features were manually input. The features were selected based on an explanation-based approach using an initial primitive model wherein important features were iteratively chosen through the decision-tree model. Alternatively, the authors also used a deep learning-based model: a convolutional neural network (CNN) with long short-term memory (LSTM) architecture that emphasizes past patterns in forecasting future data points. The CNN-LSTM model is more of a black box where the features considered important may or may not be clinically meaningful. The decision-tree-based model consistently outperformed the CNN-LSTM models across both the 4-hour and 8-hour prior-to-bedtime models. A similar study by Sathyanarayana [7] in adolescents using only 2 days of data indicated however that the CNN-based approach outperformed other machine learning models. Whereas the CNN-LSTM model could have benefited from some feature engineering as in the case of the decision-tree models is not addressed. Nevertheless, this study as others in the field stresses the importance of feature engineering in machine learning models.

It was worth noting that of the features that were significantly important, 5/18 were related to sleep efficiency on the nights prior to the final night, reaffirming the notion that machine learning models recognize patterns that are likely to be more consistent rather than inconsistent. Here too the models performed significantly poorly for individuals with regularly irregular sleep efficiency in the 5 days prior to the final night. The authors note that the models performed consistently better with weekday versus weekend patterns which is not surprising as it is reported that the UK biobank individuals' actigraphy patterns are substantially different on weekdays versus weekends [8]. The study does not address the trajectory of patterns however and one could ask whether the weekend versus weekday could have been coded as a feature.

The authors also confirmed previous findings relating to the activity prior to sleep, that is, 4 to 8 hours prior to bedtime, and sleep efficiency, albeit with a surprising one. Several studies have observed that physical exercise, defined as high-intensity activity, synergistically interacts with sleep to improve the detrimental effects of sleep loss [9]. Most of the studies note that the timing of exercise is crucial to the effect observed such that high-intensity activity prior to bedtime may be harmful to sleep. Here, the authors note a U-shaped relationship between the level of activity 4 to 8 hours prior to bedtime and low sleep efficiency. Surprisingly, the authors found that higher activity pre-sleep was associated with the likelihood of better sleep efficiency. Whether this is due to good quality sleepers in the cohort as argued by the authors or due to low/moderate levels of activity is not addressed. While the machine learning models indirectly amalgamated this information in predicting lower sleep efficiency, converting this insight into an actionable insight would require additional information about the level of activity in relation to average activity or a threshold for the given user such that an alert can be provided to diminish the level of activity in order to achieve optimal sleep.

As is the case with any machine learning model, the findings in this study are limited to the dataset chosen. Although the findings are similar to prior literature across different datasets in terms of the associations between physical activity and sleep quality, the reader is cautioned that the findings here are limited to this dataset and generalizability would require an external dataset that was not used for either development or validation in this study. Furthermore, whether there is a category of irregular sleep patterns that is beyond the pattern recognition capabilities of machine learning remains unknown. The study also did not address other modifiable behaviors such as smoking, alcohol, and caffeine intake, and their relation to predicted sleep efficiency. These are known factors that can modify sleep quality and whether their addition as features to the existing models improve the predictive ability or not remains to be seen. The study uses 5 days of activity data to infer on a single metric in the target night. An alternative approach would have been to develop predictive models that are similar to online learning models; models that dynamically tune based on the availability of additional data. The authors acknowledge that fewer than 5 days of data may provide an imperfect model, however, the addition of more than 5 days of data provides a better model remains to be seen.

The study by Sakai et al. provides a crucial first step toward the aforementioned goal of personalized recommendations for physical activity to improve overall sleep health. Transforming the output of machine learning (ML) models to actionable insights and then tracking whether those who modify their physical activity do indeed end up achieving higher sleep efficiency can provide additional confidence to a models predictive ability and is a necessary next step for translating these findings into a system that can eventually help achieve the goal of optimal sleep health using passively collected wearable data.
